# Odontographic Society of Pennsylvania

**Published:** 1863-07

**Authors:** 


### ODONTOGRAPHIC SOCIETY OF PENNSYLVANIA.

A new Association has been recently formed in Philadelphia, with the above name. There is a report of its proceedings in the July No. of the Cosmos, from which we infer it is operating in the right direction, and from a personal acquaintance with many of its members we know that whatever they undertake will be well done. T.

